# Goals, cheers, and gamma-GT: Do football tournaments affect laboratory parameters?

**DOI:** 10.3389/fpubh.2026.1839877

**Published:** 2026-06-04

**Authors:** Marlene Hollenstein, Van-Lin Nguyen, Thomas Szekeres, Klaus G. Schmetterer

**Affiliations:** Department of Laboratory Medicine, Medical University of Vienna, Vienna, Austria

**Keywords:** alcohol-related biomarkers, football tournaments, laboratory medicine, liver enzymes, mass gatherings, population health

## Abstract

**Background:**

Major international football tournaments are mass gathering events associated with increased alcohol consumption, emotional stress, and healthcare utilization. While acute health effects have been described in emergency settings, their impact on routine laboratory parameters at the population level remains unclear.

**Methods:**

We conducted a retrospective analysis of 564,316 laboratory measurements obtained within predefined observation windows surrounding 19 UEFA European Championships and FIFA World Cups (2004–2023) at a large tertiary care center. Event periods (tournament days) were compared with temporally adjacent reference periods. Primary outcomes included liver-related parameters (ALAT, ASAT, GGT), with secondary analyses of inflammatory markers (CRP, WBC) and exploratory assessment of pancreatic enzymes. Outcomes were evaluated as proportions above the upper limit of normal (ULN) and distributional measures. Statistical comparisons were performed using χ^2^ and Wilcoxon rank-sum tests, with false discovery rate correction applied as a sensitivity analysis.

**Results:**

Proportions of values exceeding the ULN were comparable between event and reference periods for ALAT (15.9% vs. 16.3%, *p* < 0.001), ASAT (11.4% vs. 11.7%, *p* = 0.031), and GGT (26.3% vs. 27.0%, p < 0.001), with only minimal absolute differences. CRP was slightly lower during event periods (40.1% vs. 41.0%, *p* < 0.001), while WBC showed no difference (24.4% vs. 24.4%, *p* = 0.93). Distributional analyses demonstrated largely overlapping patterns without systematic shifts. No consistent effects were observed across tournament context, including time zone differences, match relevance, or tournament phase. False discovery rate correction did not materially alter the interpretation of the findings.

**Conclusion:**

Despite strong behavioral and biological plausibility, major football tournaments were not associated with clinically meaningful changes in routine laboratory parameters at the population level. These findings reveal population-level buffering of event-related behavioral effects in routine laboratory data, demonstrating methodological limits of opportunistic surveillance for subtle mass gathering impacts and highlighting epidemiological resilience to transient exposures.

## 1 Introduction

Major international football tournaments such as the UEFA European Championships and FIFA World Cups represent quintessential mass gathering events, drawing billions of viewers worldwide and shaping collective behavior on a global scale. These events are associated with distinct public health challenges, driven by large-scale social dynamics including heightened emotional stress, increased alcohol consumption, altered daily routines, and shifts in healthcare utilization patterns.

Emotional stress during high-stakes matches has been linked to measurable acute health effects. During the 2006 FIFA World Cup, cardiovascular events more than doubled in association with emotionally intense matches, particularly among individuals with pre-existing risk factors (1, 2). In parallel, observational data from the UEFA EURO 2016 tournament demonstrated a substantial increase in alcohol- and injury-related emergency department visits, with a reported rise of approximately 40% during the tournament period (3, 4). These findings underscore the capacity of mass sporting events to induce acute, population-level health effects through behavioral and psychosocial mechanisms.

Alcohol consumption represents a key mediator of such effects. Globally, alcohol accounts for approximately 5% of all deaths, with heavy episodic drinking contributing disproportionately to acute health risks (5). Sporting events and holidays are well-established triggers for episodic drinking behavior in the general population (6). Biochemically, acute alcohol intake is associated with transient elevations in liver enzymes, including alanine aminotransferase (ALAT), aspartate aminotransferase (ASAT), and particularly gamma-glutamyl transferase (GGT), which reflects alcohol-induced microsomal enzyme activity and is sensitive to sustained intake above moderate thresholds (7–9).

In addition to alcohol-related effects, acute physiological stress and behavioral changes may influence laboratory parameters through other pathways. Physical exertion and stress responses have been shown to transiently elevate transaminases, while systemic inflammatory markers such as C-reactive protein (CRP) and white blood cell count (WBC) may reflect broader changes in health status and healthcare utilization during such events (9, 10).

Despite consistent evidence for increased emergency care utilization during major sporting events, the extent to which these effects translate into measurable biochemical changes at the population level remains unclear. Most existing studies focus on acute clinical outcomes, whereas routine laboratory data offer the opportunity to capture more subtle, population-wide physiological signals.

This approach builds on prior work demonstrating that large-scale laboratory datasets can detect temporally structured health patterns. In a recent long-term analysis, we observed clear holiday-associated increases in pancreatic enzyme levels, consistent with behaviorally driven fluctuations in disease activity (11). These findings support the concept of laboratory data as a sensitive tool for monitoring population-level health dynamics.

In the present study, we aimed to assess whether major international football tournaments are associated with measurable changes in routine laboratory parameters in a large, unselected patient population. We focused primarily on liver-related markers (ALAT, ASAT, GGT) as indicators of alcohol-related and behavioral effects, with inflammatory markers (CRP, WBC) and pancreatic enzymes included as secondary and exploratory outcomes.

## 2 Materials and methods

### 2.1 Study design and data source

We conducted a retrospective, laboratory-based observational study using routinely collected laboratory data from the Department of Laboratory Medicine at the Medical University of Vienna (AKH Wien), Austria’s largest university hospital and one of Europe’s major tertiary care centers. The analysis was performed at the patient-day level and followed a deterministic, reproducible workflow focused on temporal and event-associated pattern assessment. All analyses were conducted on fully anonymized datasets derived from routine laboratory information system exports. Original laboratory data containing personal identifiers were stored on secure institutional servers, and all analytical workflows were performed exclusively on anonymized datasets.

### 2.2 Study population definition and data preprocessing

**Figure 1:**
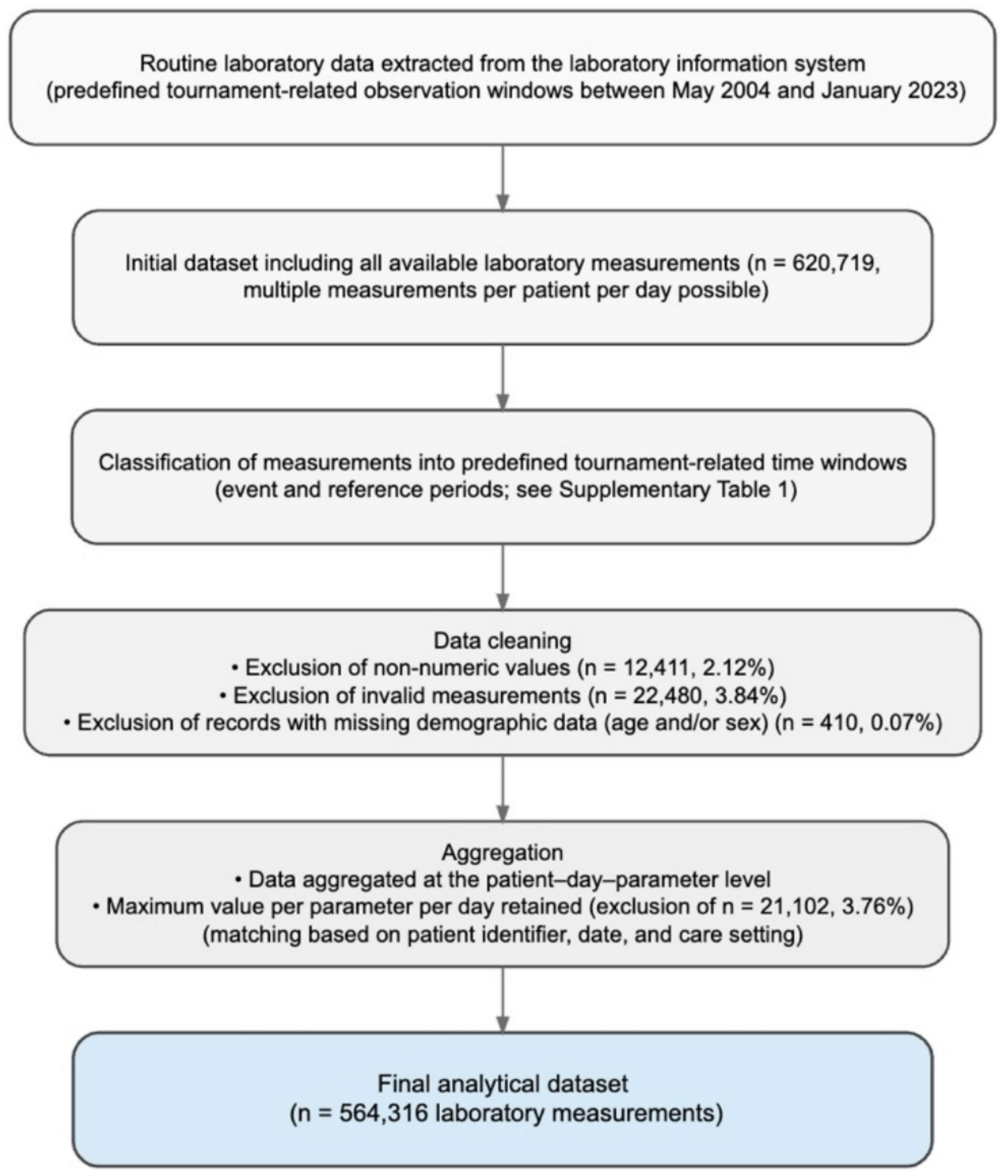
Workflow of data extraction, cleaning, aggregation, and derivation of the final analytical dataset for tournament-related laboratory measurements. Routine laboratory data were extracted from the laboratory information system within predefined tournament-related observation windows, classified into event and reference periods, cleaned by exclusion of non-numeric, invalid, and incomplete records, aggregated at the patient-day-parameter level using the maximum daily value per parameter, and reduced to the final analytical dataset of 564,316 measurements.

Laboratory measurements were included from predefined observation windows covering major international football tournaments between May 2004 and January 2023. These windows comprised the full tournament periods (event periods) as well as predefined reference periods before and after each tournament. Measurements originated from different clinical settings, including the emergency department (ED), trauma care, and other ambulatory services, reflecting a broad spectrum of healthcare utilization. Raw laboratory data were processed using a deterministic pipeline. Measurements were aggregated at the patient-day-parameter level, retaining the maximum value per parameter per day to capture peak biochemical activity, and subsequently structured into a wide-format dataset. Non-numeric or invalid values were excluded. A detailed overview of the data extraction, preprocessing, and aggregation workflow is provided in Figure 1.

For each tournament, the event period was defined as the full duration from the first to the last tournament day, including days without matches. Reference periods were defined *a priori* as the 30 calendar days preceding the tournament start and the 30 calendar days following the tournament end, ensuring temporal proximity and seasonal comparability (Supplementary Table 1). Within event periods, match days were defined as calendar days on which at least one match was played, allowing distinction between tournament days with and without direct match exposure.

### 2.3 Event and reference period definition

For each tournament, the event period was defined as the full tournament duration, from the first to the last tournament day, irrespective of whether matches were played on every calendar day. Thus, all calendar days within the tournament period were classified as event days. Reference periods were defined *a priori* as the 30 calendar days preceding the tournament start and the 30 calendar days following the tournament end. These windows were selected to provide temporally adjacent comparison periods while preserving seasonal comparability. A detailed overview of all tournament and reference windows is provided in Supplementary Table 1. Separately, match days were defined as calendar days within the event period on which at least one tournament match was played. This distinction allowed comparison between tournament days with and without direct match exposure.

### 2.4 Laboratory parameters

A broad panel of routinely measured laboratory parameters was available, including markers of liver function, systemic inflammation, pancreatic enzymes, renal function, and metabolic status. Primary analyses focused on liver-related parameters—alanine aminotransferase (ALAT), aspartate aminotransferase (ASAT), and gamma-glutamyl transferase (GGT)—based on *a priori* hypotheses regarding potential behavioral and alcohol-related effects during football events. Secondary analyses included C-reactive protein (CRP) and white blood cell count (WBC) as markers of systemic inflammation and acute illness burden. Pancreatic enzyme measurements (lipase, amylase, and pancreatic amylase) were included as exploratory outcomes to assess whether potential event-associated effects extend beyond liver-related markers. For ALAT, ASAT, and GGT, changes were regarded as analytically meaningful only if they exceeded the expected analytical imprecision of the Roche cobas platform or crossed the manufacturer’s stated interference/recovery limits (12–14).

### 2.5 Outcome definitions and statistical analysis

Upper limits of normal (ULN) were defined based on institutional reference ranges. For each measurement, the ratio to ULN was calculated. Primary outcomes included the proportion of values exceeding the ULN and the distribution of ULN ratios. For selected parameters, severity categories were defined (normal, mild, moderate, severe) based on multiples of ULN.

In the context of large-scale routine laboratory data, the analytical approach was designed to detect consistent population-level signals rather than transient or individual-level fluctuations. Accordingly, the study followed a signal-to-noise framework, assuming that only sustained and sufficiently large effects across the population would produce detectable changes in aggregate laboratory parameters. Based on physiological considerations, we hypothesized that football tournament exposure would be associated with small but directionally consistent upward shifts in liver-related markers during higher-exposure periods (e.g., match days, evening hours, and later tournament stages). Acute changes were expected for ALAT and ASAT, whereas more cumulative changes were expected for GGT. These hypotheses guided the selection of primary and secondary analyses, including comparisons across event periods, match days, and predefined temporal strata. Changes were considered meaningful only if they exceeded expected analytical variation or crossed predefined ULN or severity thresholds. Analytical imprecision of the Roche cobas platform (Roche CVs 1.3–3.7%) was considered when interpreting observed differences (12–14).

Analyses were primarily descriptive and pattern oriented. Distributional differences between event and reference periods were assessed using non-parametric methods, and categorical comparisons were performed using χ^2^ or Fisher’s exact tests, as appropriate. Laboratory values were analyzed both as continuous variables (ratio to ULN) and as categorical outcomes (above vs. below ULN), with additional severity stratification for selected parameters. Stratified analyses were performed by care setting and tournament type. Given the exploratory and descriptive nature of the analyses, statistical significance was interpreted in conjunction with effect size, consistency, and clinical relevance. As a sensitivity analysis for multiple comparisons, Benjamini–Hochberg false discovery rate (FDR) correction was applied to all inferential *p*-values. FDR-adjusted q-values were interpreted descriptively alongside effect sizes, consistency of direction, and clinical or analytical relevance. The primary interpretation remained based on absolute differences, distributional patterns, and predefined clinical or analytical thresholds rather than statistical significance alone.

Formal lag-based time-series models were not applied because tournament exposure was temporally dense and overlapping, particularly during group stages with multiple matches per day and consecutive match days. In addition, individual exposure timing was unavailable and laboratory testing was clinically driven rather than scheduled relative to match exposure. Therefore, short-term effects were assessed using predefined temporal contrasts, including match days versus non-match days, time-of-day strata, prime-time sampling, and tournament phase analyses.

All analyses were conducted using R version 4.5.2 (R Foundation for Statistical Computing).

### 2.6 Ethics approval

According to institutional review board guidance, analyses of fully anonymized retrospective laboratory data without re-identification risk do not require individual informed consent. The study was approved by the Ethics Committee of the Medical University of Vienna (EK2239/25), which confirmed that the use of fully anonymized retrospective laboratory data did not require individual informed consent.

## 3 Results

### 3.1 Study population

The analysis comprised 564,316 laboratory measurements obtained during predefined tournament-related observation windows, including 181,718 measurements during event periods and 382,598 during reference periods. Median age was identical between groups (54 years [IQR 37–66], Minimum to Maximum age 0–115 years in the reference period compared to 0–113 years during the event period). The sex distribution was balanced, with 50.3% male and 49.7% female individuals in both event and reference periods. The majority of measurements originated from ambulatory care settings (89.0% during event periods vs. 88.8% during reference periods), followed by the emergency department (9.3% vs. 9.8%) and trauma care (1.7% vs. 1.5%). Overall, demographic characteristics and care setting distribution were highly comparable between event and reference periods (Table 1).

**Table 1:** Study population characteristics.

Characteristic	Reference days	Event day
Observations, *n*	382,598	181,718
Age, median (IQR / Min-Max)	54.0 (37.0–66.0/0–115.0)	54.0 (37.0–66.0/0–113.0)
Male, *n* (%)	191,848 (50.3%)	90,870 (50.3%)
Female, *n* (%)	189,703 (49.7%)	89,774 (49.7%)
Emergency department, *n* (%)	37,326 (9.8%)	16,951 (9.3%)
Other ambulatory care, *n* (%)	339,706 (88.8%)	161,720 (89.0%)
Trauma surgery, *n* (%)	5,566 (1.5%)	3,047 (1.7%)

Overall dataset: 564,316 observations across 887 dates (2004-05-13 to 2023-01-17). Values are presented as *n* (%), unless otherwise stated. Age is shown as median (IQR).

### 3.2 Primary laboratory findings during event and reference periods

Primary analyses focused on liver-related parameters (ALAT, ASAT, and GGT), selected based on *a priori* hypotheses regarding potential behavioral and alcohol-related effects during major football events.

Across all three parameters, the proportion of values exceeding the upper limit of normal (ULN) was highly comparable between event and reference periods (Table 2). For ALAT, proportions were 15.9% during event periods versus 16.3% during reference periods (*Δ* − 0.5 percentage points; *p* < 0.001; q = 0.002). For ASAT, corresponding values were 11.4% versus 11.7% (Δ − 0.3 percentage points; *p* = 0.031; q = 0.054), and for GGT 26.3% versus 27.0% (Δ − 0.6 percentage points; *p* < 0.001; q = 0.002).

**Table 2:** Comparison of primary and secondary laboratory parameters between event and reference periods.

Parameter	*n* (reference)	*n* (event)	>ULN (reference), *n* (%)	>ULN (event), *n* (%)	Δ (%-points)	Ratio to ULN (reference), median (IQR)	Ratio to ULN (event), median (IQR)	*p*-value (>ULN)	q-value (FDR, >ULN)	*p*-value (ratio)	q-value (FDR, ratio)
ALAT	232,142	111,066	37,891 (16.3%)	17,594 (15.9%)	−0.5	0.54 (0.40–0.82)	0.54 (0.40–0.80)	<0.001	0.002	<0.001	0.002
ASAT	228,467	109,511	26,588 (11.7%)	12,456 (11.4%)	−0.3	0.58 (0.46–0.76)	0.57 (0.46–0.74)	0.031	0.054	0.001	0.002
CRP	213,195	103,096	87,320 (41.0%)	41,356 (40.1%)	−0.8	0.70 (0.26–2.10)	0.68 (0.26–2.02)	<0.001	0.002	<0.001	0.002
GGT	223,270	107,143	60,123 (27.0%)	28,180 (26.3%)	−0.6	0.58 (0.38–1.08)	0.58 (0.37–1.06)	<0.001	0.002	<0.001	0.002
WBC	256,659	122,078	62,420 (24.4%)	29,658 (24.4%)	0	0.76 (0.58–0.99)	0.76 (0.58–0.99)	0.928	0.928	0.877	0.906

Values are presented as *n* (%) or median (IQR). Δ represents the absolute difference in percentage points (event minus reference). ULN = upper limit of normal. *p* values for proportions above ULN were calculated using χ^2^ or Fisher’s exact test, and for continuous variables using the Wilcoxon rank-sum test. q-values were calculated using Benjamini–Hochberg false discovery rate correction.

**Figure 2:**
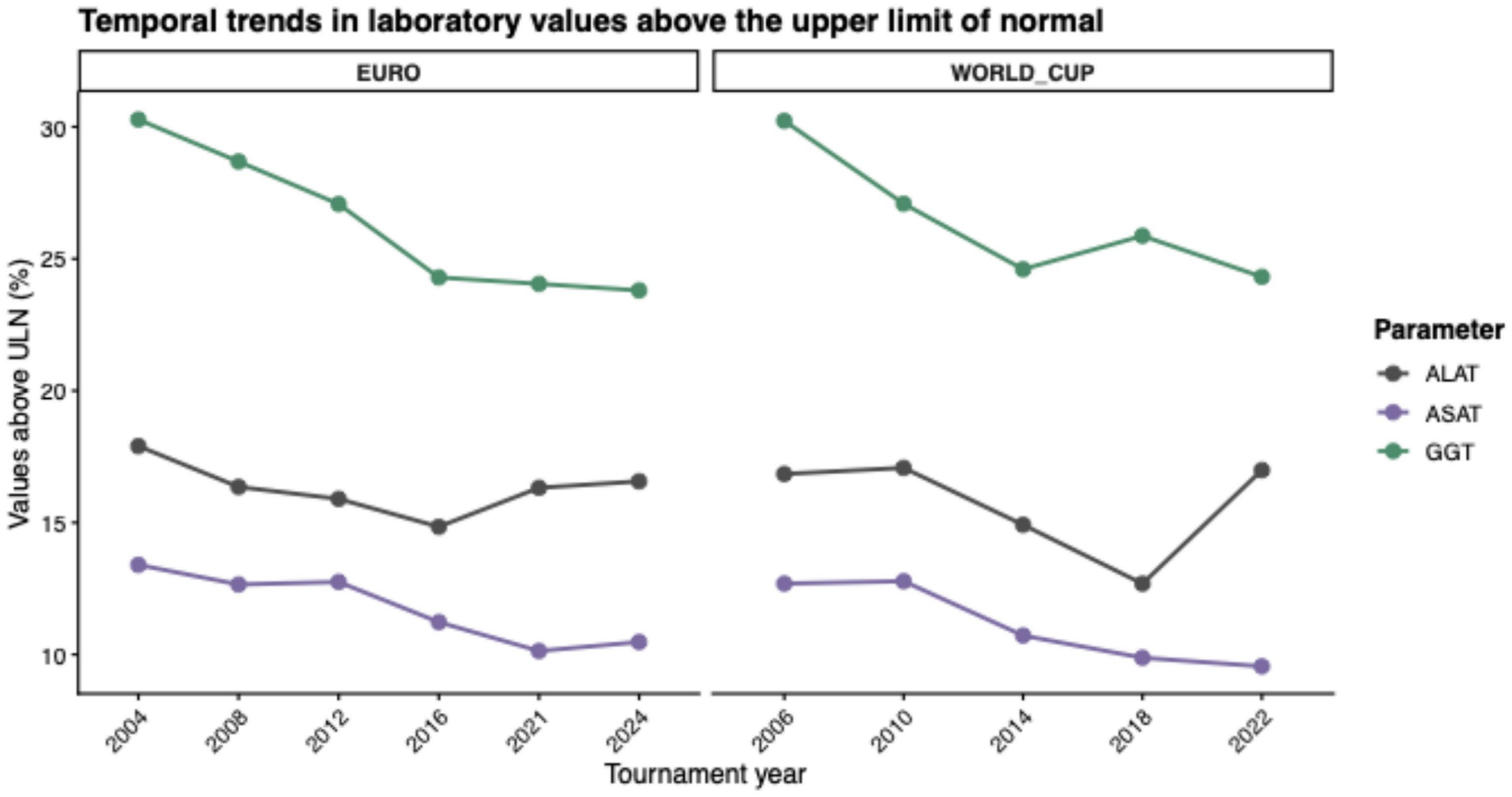
Proportions of laboratory values exceeding the upper limit of normal (ULN) for ALAT, ASAT, and GGT. The parameters are shown across individual football tournaments from 2004 to 2024. Values are shown by tournament year and illustrate stable patterns over time without consistent event-associated increases.

Analysis of proportions of laboratory values exceeding the upper limit of normal (ULN) across tournaments showed largely stable patterns over time, without consistent increases during event periods (Figure 2). Median values remained well below the ULN for all parameters (ALAT: 0.54 vs. 0.54; ASAT: 0.57 vs. 0.58; GGT: 0.58 vs. 0.58), with only minimal differences that did not follow a consistent direction.

Assessment of severity distributions further supported these findings. Across all liver parameters, the majority of values were within the normal range, with only a small proportion of moderate or severe elevations (Table 3). Statistically significant differences in severity distribution were observed for ALAT (*p* = 0.006; q = 0.011) and GGT (*p* < 0.001; q = 0.002), whereas ASAT did not reach statistical significance after FDR correction (*p* = 0.059; q = 0.095). Importantly, these differences reflected only minimal shifts toward slightly higher proportions of normal values during event periods, without any increase in moderate or severe abnormalities.

**Table 3:** Severity distribution of primary liver parameters on event days versus reference days.

Parameter	Reference (normal)	Reference (mild)	Reference (moderate)	Reference (severe)	Event (normal)	Event (mild)	Event (moderate)	Event (severe)	*p*-value	q-value (FDR)
ALAT	216,499 (83.6%)	31,074 (12.0%)	9,090 (3.5%)	2,201 (0.9%)	104,832 (84.1%)	14,583 (11.7%)	4,227 (3.4%)	1,069 (0.9%)	0.006	0.011
ASAT	225,162 (88.5%)	21,770 (8.6%)	6,030 (2.4%)	1,513 (0.6%)	108,879 (88.7%)	10,385 (8.5%)	2,748 (2.2%)	734 (0.6%)	0.059	0.095
GGT	182,698 (73.3%)	36,525 (14.7%)	20,313 (8.1%)	9,707 (3.9%)	88,892 (73.9%)	17,307 (14.4%)	9,563 (8.0%)	4,444 (3.7%)	<0.001	0.002

Severity categories were defined relative to the upper limit of normal (ULN): Normal, ≤ULN; Mild, >1 to 2 × ULN; Moderate, >2 to 5 × ULN; Severe, >5 × ULN. *p*-values were obtained using chi-square tests for the overall severity distribution. q-values were calculated using Benjamini–Hochberg false discovery rate correction.

Stratified analyses by clinical setting demonstrated consistent findings across emergency department, ambulatory care, and trauma settings (Table 4). Within each setting, proportions above ULN and distributional measures were comparable between event and reference periods. While some comparisons reached statistical significance in larger subgroups, absolute differences remained small (generally ≤1 percentage point), and no consistent directional pattern was observed.

**Table 4:** Event days versus reference days stratified by clinical setting.

Setting	Parameter	*n* (reference)	*n* (event)	>ULN (reference), *n* (%)	>ULN (event), *n* (%)	Δ (%-points)	Ratio to ULN (reference), median (IQR)	Ratio to ULN (event), median (IQR)	*p*-value (>ULN)	q-value (FDR, >ULN)	*p*-value (ratio)	q-value (FDR, ratio)
Emergency department	ALAT	39,287	17,979	6,377 (16.2%)	2,842 (15.8%)	−0.4	0.54 (0.40–0.82)	0.54 (0.40–0.80)	0.204	0.257	0.125	0.174
Emergency department	ASAT	38,716	17,726	4,938 (12.8%)	2,142 (12.1%)	−0.7	0.60 (0.48–0.77)	0.60 (0.48–0.77)	0.027	0.049	0.391	0.465
Emergency department	CRP	34,815	16,090	17,656 (50.7%)	8,151 (50.7%)	−0.1	1.06 (0.30–4.76)	1.04 (0.30–4.94)	0.916	0.928	0.666	0.723
Emergency department	GGT	34,269	15,866	8,268 (24.1%)	3,718 (23.4%)	−0.7	0.53 (0.35–0.98)	0.52 (0.35–0.97)	0.093	0.136	0.037	0.061
Emergency department	WBC	38,184	17,500	16,963 (44.4%)	7,888 (45.1%)	0.6	0.95 (0.75–1.23)	0.96 (0.75–1.23)	0.159	0.209	0.219	0.271
Other ambulatory care	ALAT	214,089	103,685	34,917 (16.3%)	16,496 (15.9%)	−0.4	0.54 (0.40–0.82)	0.54 (0.40–0.80)	0.004	0.008	<0.001	0.002
Other ambulatory care	ASAT	210,401	102,119	23,181 (11.0%)	11,081 (10.9%)	−0.2	0.57 (0.46–0.74)	0.57 (0.46–0.74)	0.189	0.243	0.033	0.056
Other ambulatory care	CRP	199,068	97,164	76,774 (38.6%)	36,635 (37.7%)	−0.9	0.64 (0.24–1.84)	0.62 (0.24–1.76)	<0.001	0.002	<0.001	0.002
Other ambulatory care	GGT	209,694	101,565	56,801 (27.1%)	26,871 (26.5%)	−0.6	0.58 (0.38–1.08)	0.58 (0.37–1.07)	<0.001	0.002	<0.001	0.002
Other ambulatory care	WBC	241,430	115,852	49,465 (20.6%)	23,582 (20.5%)	−0.1	0.73 (0.56–0.94)	0.73 (0.56–0.94)	0.414	0.483	0.697	0.744
Trauma surgery	ALAT	5,798	3,198	1,071 (18.5%)	541 (16.9%)	−1.6	0.54 (0.40–0.86)	0.56 (0.40–0.83)	0.068	0.107	0.494	0.562
Trauma surgery	ASAT	5,674	3,138	1,194 (21.1%)	644 (20.5%)	−0.5	0.66 (0.52–0.94)	0.66 (0.50–0.91)	0.574	0.634	0.083	0.124
Trauma surgery	CRP	4,872	2,660	2,128 (43.7%)	1,106 (41.6%)	−2.1	0.76 (0.26–2.84)	0.70 (0.26–2.50)	0.083	0.124	0.143	0.192
Trauma surgery	GGT	5,526	2,882	1,476 (26.7%)	725 (25.2%)	−1.6	0.57 (0.35–1.07)	0.55 (0.35–1.02)	0.127	0.174	0.100	0.143
Trauma surgery	WBC	5,840	3,187	3,155 (54.1%)	1,758 (55.2%)	1.1	1.04 (0.80–1.38)	1.04 (0.82–1.37)	0.324	0.393	0.500	0.562

Values are presented as *n* (%) or median (IQR). Δ represents the absolute difference in percentage points (event minus reference). ULN = upper limit of normal. *p*-values for proportions above ULN were calculated using χ^2^ or Fisher’s exact test, and for continuous variables using the Wilcoxon rank-sum test. q-values were calculated using Benjamini–Hochberg false discovery rate correction.

### 3.3 Secondary laboratory outcomes

Secondary analyses included WBC and CRP as markers of systemic inflammation and acute illness burden. CR *p* values above the ULN were slightly lower during event periods compared with reference periods (40.1% vs. 41.0%; *Δ* − 0.8 percentage points; *p* < 0.001; q = 0.002), while WBC showed no difference (24.4% vs. 24.4%; Δ 0.0 percentage points; *p* = 0.928; q = 0.928).

Pancreatic enzyme parameters (lipase, amylase, and pancreatic amylase) were evaluated as exploratory outcomes. Across all pancreatic parameters, no relevant differences between event and reference periods were observed. Both proportions above ULN and distributional measures remained comparable, without evidence of a shift toward higher values during event periods.

Additional laboratory parameters, including markers of renal function, hematologic indices, and metabolic status, were analyzed descriptively. No consistent or clinically meaningful differences between event and reference periods were observed across these parameters.

Additional sensitivity analyses were performed to assess short-term and context-specific effects within tournament periods. Comparisons of match days versus non-match days showed no consistent differences across parameters or time-of-day strata (Supplementary Table 2). Stratification by time of day demonstrated that laboratory value distributions were primarily driven by sampling patterns, with largely overlapping medians between event and reference periods across all time windows (Supplementary Table 3). Further analyses focusing on periods of increased exposure, including prime-time sampling, knockout-stage matches, and matches involving the Austrian national team, similarly showed no consistent or clinically meaningful differences in laboratory parameters (Supplementary Table 4–5).

**Figure 3:**
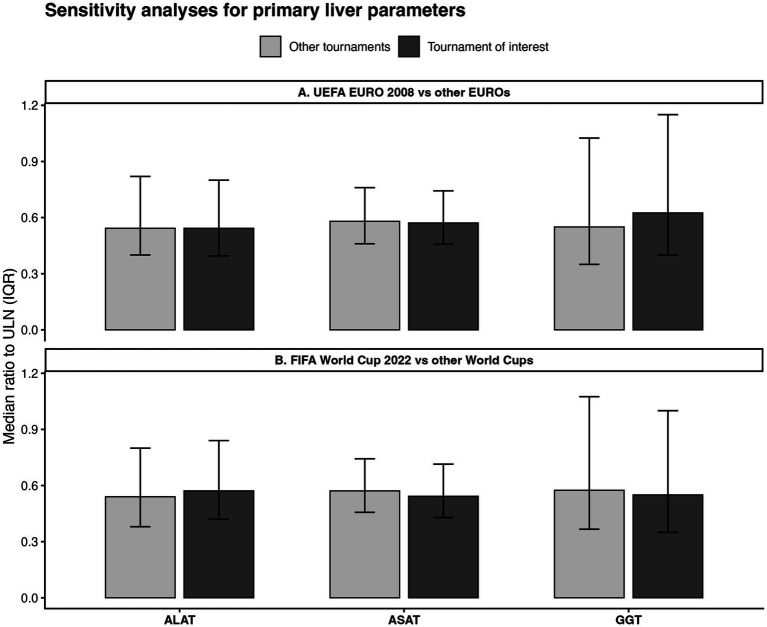
Sensitivity analyses for primary liver parameters. **(A)** Comparison of median ratios to the upper limit of normal (ULN) for alanine aminotransferase (ALAT), aspartate aminotransferase (ASAT), and gamma-glutamyl transferase (GGT) during UEFA EURO 2008 (tournament of interest) versus other EURO tournaments. **(B)** Comparison of median ratios to ULN during the FIFA World Cup 2022 (tournament of interest) versus other World Cups. Bars represent median values with interquartile ranges (IQR). Across both analyses, no consistent or clinically meaningful differences were observed between tournaments of interest and corresponding comparison groups.

Analyses of specific tournament contexts were performed to assess whether particular event characteristics, including increased public engagement or atypical scheduling, were associated with differential laboratory patterns (Table 5). For the UEFA Euro 2008, representing a home tournament setting, slightly higher proportions above the ULN were observed for GGT (28.8% vs. 25.9%, *Δ* + 2.9 percentage points; *p* < 0.001; q = 0.002) and ASAT (12.6% vs. 11.3%, Δ + 1.2 percentage points; *p* < 0.001; q = 0.002), while no difference in proportions above ULN was observed for ALAT (*p* = 0.767; q = 0.805). Despite statistical significance in selected comparisons, absolute differences remained small, and no corresponding shift toward higher severity categories was observed. For the FIFA World Cup 2022, which was conducted during winter months with altered match timing, proportions above the ULN were slightly lower for ASAT (9.6% vs. 11.8%, *Δ* − 2.1 percentage points; *p* < 0.001; q = 0.002) and GGT (25.2% vs. 27.0%, Δ − 1.9 percentage points; *p* < 0.001; q = 0.002), while ALAT showed a minimal increase (16.8% vs. 15.6%, Δ + 1.2 percentage points; *p* < 0.001; q = 0.002). Again, distributional analyses did not indicate a consistent shift toward higher values. These findings were consistent when examining continuous distributions relative to ULN, which showed largely overlapping median values between tournaments of interest and comparison groups (Figure 3).

**Table 5:** Sensitivity analyses by tournament edition.

Comparison	Parameter	*n* (comparator)	*n* (tournament of interest)	>ULN (comparator), *n* (%)	>ULN (tournament of interest), n (%)	Δ (%-points)	Ratio to ULN (comparator), median (IQR)	Ratio to ULN (tournament of interest), median (IQR)	*p*-value (>ULN)	q-value (FDR, >ULN)	*p*-value (ratio)	q-value (FDR, ratio)
Other EUROs vs EURO 2008	ALAT	177,689	31,617	29,315 (16.5%)	5,242 (16.6%)	0.1	0.54 (0.40–0.82)	0.54 (0.40–0.82)	0.767	0.805	0.009	0.017
Other EUROs vs EURO 2008	ASAT	174,535	31,291	19,783 (11.3%)	3,936 (12.6%)	1.2	0.57 (0.46–0.76)	0.58 (0.46–0.77)	<0.001	0.002	<0.001	0.002
Other EUROs vs EURO 2008	CRP	153,113	31,429	62,574 (40.9%)	13,235 (42.1%)	1.2	0.68 (0.24–2.10)	0.74 (0.28–2.14)	<0.001	0.002	<0.001	0.002
Other EUROs vs EURO 2008	GGT	171,193	30,184	44,301 (25.9%)	8,703 (28.8%)	2.9	0.57 (0.35–1.05)	0.62 (0.40–1.15)	<0.001	0.002	<0.001	0.002
Other EUROs vs EURO 2008	WBC	194,055	34,638	46,650 (24.2%)	7,842 (22.7%)	−1.5	0.76 (0.58–0.99)	0.74 (0.56–0.97)	<0.001	0.002	<0.001	0.002
Other World Cups vs World Cup 2022	ALAT	140,463	34,267	21,929 (15.6%)	5,758 (16.8%)	1.2	0.54 (0.40–0.80)	0.57 (0.40–0.83)	<0.001	0.002	<0.001	0.002
Other World Cups vs World Cup 2022	ASAT	138,615	33,333	16,263 (11.8%)	3,198 (9.6%)	−2.1	0.58 (0.46–0.76)	0.54 (0.43–0.71)	<0.001	0.002	<0.001	0.002
Other World Cups vs World Cup 2022	CRP	137,215	32,912	54,193 (39.5%)	12,448 (37.8%)	−1.7	0.66 (0.26–1.98)	0.60 (0.22–2.00)	<0.001	0.002	<0.001	0.002
Other World Cups vs World Cup 2022	GGT	135,227	33,198	36,501 (27.0%)	8,354 (25.2%)	−1.9	0.58 (0.38–1.08)	0.55 (0.35–1.02)	<0.001	0.002	<0.001	0.002
Other World Cups vs World Cup 2022	WBC	156,485	36,815	38,628 (24.7%)	9,691 (26.4%)	1.7	0.76 (0.58–1.00)	0.78 (0.59–1.02)	<0.001	0.002	<0.001	0.002

Values are presented as *n* (%) or median (IQR). Δ represents the difference in percentage points (event minus reference). ULN = upper limit of normal. *p*-values for proportions above ULN were calculated using χ^2^ or Fisher’s exact test, and for continuous variables using the Wilcoxon rank-sum test. q-values were calculated using Benjamini–Hochberg false discovery rate correction.

Across both sensitivity analyses, findings remained directionally inconsistent and of small magnitude, without evidence of clinically meaningful changes in laboratory patterns. FDR sensitivity analysis did not materially alter the interpretation of the findings. 63 inferential comparisons were evaluated; 38 were nominally significant at *p* < 0.05, and 35 remained significant after Benjamini–Hochberg FDR correction (q < 0.05). However, findings that remained significant after FDR correction were small in magnitude, directionally inconsistent, and below clinical or analytical relevance thresholds.

Heterogeneity analyses consistently supported the main findings of no clinically meaningful event effects. Match vs. non-match days (Supplementary Table 2), time-of-day stratification (Supplementary Table 3), group vs. knockout stages (Supplementary Table 4), and Austria vs. other matches (Supplementary Table 5) all showed minimal median differences (≤0.02 ULN ratios) without directional consistency. These results did not support the expectation of amplified effects during high-exposure scenarios (e.g., prime-time, national matches) and reinforce the primary conclusion that tournament-related changes are analytically negligible across contexts.

### 3.4 Conclusion

Across all analyses, no consistent or clinically meaningful differences in laboratory parameters were observed between event and reference periods. While several comparisons reached statistical significance, these were small in magnitude and did not follow a consistent directional pattern. Importantly, no evidence of a shift toward more severe laboratory abnormalities was detected, and findings were consistent across clinical settings and sensitivity analyses. Additional stratified analyses did not reveal meaningful differences according to tournament context. In particular, no consistent variation was observed with respect to tournament location and associated time zone differences, matches involving Austria or teams with potentially increased local population interest, or across different phases of tournament intensity. These findings suggest that observed laboratory patterns are primarily driven by healthcare utilization and sampling structure rather than direct biological responses to tournament-related exposures.

## 4 Discussion

### 4.1 Principal findings

In this large retrospective analysis of over 560,000 laboratory measurements obtained during major international football tournaments, we hypothesized a short-term increase in aminotransferases following match days and more gradual increases in GGT over sustained tournament periods. Despite our hypothesis, no clinically meaningful differences in liver-related laboratory parameters were observed between event and reference periods. Across ALAT, ASAT, and GGT, proportions of values exceeding the upper limit of normal (ULN) as well as distributional measures remained highly comparable, with only minimal absolute differences despite statistical significance. Similarly, no consistent changes were detected in inflammatory markers (CRP, WBC) or pancreatic enzymes.

These findings suggest that, at a population level within routine outpatient and emergency care, major football events are not associated with measurable shifts in biochemical markers of liver injury, systemic inflammation, or pancreatic involvement.

Our null results generate two key insights beyond “no effect observed.” Methodologically, they quantify routine laboratory surveillance limits for mass gatherings, where ED signals do not propagate to population-level biomarkers despite plausibility. Epidemiologically, they demonstrate buffering: well-documented alcohol/stress behaviors are diluted across heterogeneous populations, yielding no detectable aggregate signal. This contrasts with acute ED studies and reframes mass gatherings as events with compartmentalized rather than population-wide biochemical impacts (15, 16).

### 4.2 Comparison with previous literature

Mass gathering events, including major football tournaments, are commonly associated with increased health service utilization and risk-related behaviors. Previous studies have demonstrated substantial increases in alcohol-related and injury-related emergency department visits during such events, including a reported 43% rise during UEFA EURO 2016 (17).

However, these findings primarily originate from acute care settings and focus on severe or symptomatic cases. In contrast, our analysis captures a broad, unselected population undergoing routine laboratory testing. This distinction is critical, as event-associated effects observed in emergency settings may not translate into detectable shifts at the population level. This interpretation is supported by recent evidence suggesting that increases in alcohol-related harm during mass gatherings are often concentrated in specific subgroups, while the broader population remains largely unaffected (18).

### 4.3 Alcohol-related mechanisms and biomarker considerations

Alcohol consumption represents one of the most plausible mechanisms linking football events to potential biochemical changes. Heavy episodic drinking is a well-established feature of sporting events and contributes substantially to global disease burden (5, 19). Acute alcohol intake can lead to transient elevations in liver enzymes, particularly ALAT, ASAT, and GGT (20). Among these markers, GGT is considered particularly sensitive to alcohol exposure, reflecting microsomal enzyme induction and showing detectable increases in individuals consuming more than 50 g ethanol per day (21). Despite this biological plausibility, our findings do not support a meaningful increase in alcohol-related biochemical signatures at the population level during football tournaments. Even GGT, the most sensitive marker, showed no consistent or clinically relevant shift. This may reflect the limited sensitivity of conventional biomarkers to short-term or moderate changes in alcohol consumption, as well as dilution effects in large heterogeneous populations (22).

Several factors may explain the absence of detectable laboratory changes despite known behavioral shifts during football events. First, event-related behaviors such as alcohol consumption are unevenly distributed across the population and often confined to specific subgroups, including younger individuals and highly engaged fans (23, 24). At a population level, one might nevertheless expect that repeated exposure during tournament periods—particularly in the context of prolonged viewing, public gatherings, and increasing match intensity toward later stages—could lead to a modest upward shift in baseline laboratory values, rather than isolated acute peaks. Such effects would be expected to manifest as small but consistent distributional changes across the event period. However, no such pattern was observed in the present analysis. This suggests that exposure to event-related behaviors is either not sufficiently widespread across the general population or largely restricted to subgroups, resulting in dilution of potential effects in aggregated routine laboratory data. In addition, exposure was not directly measured at the individual level but approximated using event periods, resulting in non-differential exposure misclassification that may further bias results toward the null.

Second, there is a temporal mismatch between potential exposures and laboratory measurements. Event-related behaviors such as episodic alcohol consumption or acute emotional stress typically occur over short time windows (e.g., match days or evenings), whereas laboratory measurements are obtained at clinically driven and irregular time points. Furthermore, commonly used liver enzymes, particularly GGT, reflect cumulative exposure over days to weeks rather than acute changes. For example, while aminotransferases may show transient elevations within hours to days following acute exposure, GGT typically reflects sustained exposure over longer periods, further limiting its sensitivity to short-lived behavioral changes. As a result, transient physiological responses may not be captured within routine laboratory testing. In addition, laboratory testing in routine clinical practice is driven by indication rather than systematic sampling. Parameters such as ALAT, ASAT, GGT, and CRP are frequently ordered as part of standardized diagnostic panels, reflecting symptom-driven clinical decision-making rather than targeted assessment of specific exposures. Consequently, laboratory data primarily capture underlying disease processes and healthcare utilization patterns, rather than transient, exposure-related physiological changes. Observed variations across time-of-day strata further indicate that laboratory value distributions are predominantly driven by underlying sampling patterns and healthcare utilization, rather than event-related exposures.

Third, compensatory or protective factors may play a role. Physical activity and lifestyle factors can modulate liver enzyme levels over time, potentially offsetting short-term increases (25). Finally, the discrepancy between emergency department findings and routine laboratory data likely reflects differences in case mix. While ED studies capture acute and often severe presentations, routine laboratory testing reflects a broader and less selected patient population, resulting in fundamentally different signal profiles (26).

Mass gatherings are widely recognized as complex public health phenomena, with potential impacts ranging from infectious disease transmission to behavioral risk patterns. Football tournaments represent a unique subtype characterized by prolonged duration and repeated exposure, rather than a single event (27). Despite this, our findings suggest that such events do not necessarily translate into measurable biochemical changes at the population level. This challenges assumptions that large-scale social events uniformly result in detectable health impacts across broad populations. Instead, effects may be localized, transient, or limited to specific high-risk groups.

### 4.4 Detection framework and competing explanations

Our findings highlight the detection limits of routine laboratory data within an epidemiological signal-vs-noise framework. Small true effects from event-related behaviors may be masked by dilution in heterogeneous populations, where only subgroups (e.g., heavy drinkers) show biomarker shifts. Liver enzymes like GGT have limited sensitivity for transient exposures, compounded by temporal mismatches between match-day peaks and irregular sampling. Behavioral heterogeneity further reduces signal strength, as non-responders dominate aggregate data. These factors explain the absence of robust signals despite statistical significance, emphasizing routine data’s value for surveillance but low power for subtle effects (28).

### 4.5 Contextual modifiers and robustness of findings

Importantly, the absence of a measurable laboratory signal remained consistent across multiple context-specific analyses. No meaningful differences were observed with respect to tournament location and associated time zone shifts, including tournaments held outside Europe with substantially altered match schedules. Similarly, matches involving Austria or teams with high migration population relevance—representing scenarios with potentially increased emotional engagement and viewership—did not show any detectable impact on laboratory parameters. In addition, analyses across tournament phases and varying match intensities did not reveal consistent patterns, despite the expectation that later-stage matches or high-stakes encounters would amplify behavioral responses.

Taken together, these findings indicate that even under conditions theoretically most likely to induce physiological effects—such as peak emotional engagement, altered circadian exposure, or heightened population interest—no robust biochemical signal emerges at the population level. The consistency of these findings across multiple exposure intensities and analytical stratifications suggests that the absence of detectable effects is not attributable to insufficient analytical resolution, but reflects a true lack of measurable population-level response within routine laboratory data.

### 4.6 Relation to previous work and implications

Given the well-documented increases in alcohol consumption, emotional stress, and acute healthcare utilization associated with such events, measurable biological effects at the population level would be expected (1, 2, 4, 17, 23, 24, 26, 27). However, despite this strong *a priori* plausibility, no corresponding signal was observed in routine laboratory parameters in the present analysis. The FDR sensitivity analysis further supports this interpretation: although many nominally significant comparisons remained statistically significant after correction, the corresponding effect sizes were minimal and inconsistent, underscoring that statistical robustness in very large datasets does not necessarily imply biological, analytical, or clinical relevance.

This finding challenges the assumption that large-scale social events with clear behavioral impact necessarily translate into detectable biochemical changes in unselected patient populations. Instead, it suggests that such effects may be transient, compartmentalized, or restricted to specific high-risk subgroups, and therefore not captured in aggregate laboratory data. In addition, the magnitude of observed differences was smaller than the expected analytical imprecision of the Roche cobas assay platform and did not show a consistent directional pattern, further supporting the interpretation that these variations are not analytically or clinically meaningful (29).

### 4.7 Strengths and limitations

This study benefits from a large sample size, a long observation period spanning nearly two decades, and the inclusion of both outpatient and emergency care data from a tertiary care center, allowing robust assessment of population-level patterns across diverse clinical settings. The use of predefined event and reference windows ensured temporal comparability while minimizing seasonal confounding. In addition, normalization to the upper limit of normal (ULN) enabled consistent interpretation across laboratory parameters and analytical platforms over time.

However, several limitations should be considered. First, the observational design precludes causal inference, and individual-level exposure data—such as alcohol consumption, match viewership, or behavioral changes—were not available. As a result, potential associations between football events and laboratory findings can only be assessed indirectly at the population level. Second, laboratory measurements were obtained based on clinical indications rather than systematic sampling, introducing potential selection bias. While this reflects real-world healthcare utilization, it may limit sensitivity for detecting subtle or transient effects in otherwise healthy individuals. Third, conventional laboratory biomarkers, particularly liver enzymes, may have limited sensitivity for short-term or moderate behavioral exposures, such as episodic alcohol intake or acute stress responses (21). Transient physiological changes may therefore remain undetected within routine laboratory testing frameworks. Fourth, although analyses were stratified by clinical setting, residual confounding due to differences in patient populations, healthcare access, or care-seeking behavior cannot be fully excluded. In particular, outpatient and emergency care settings capture distinct clinical spectra, which may influence baseline laboratory distributions. Formal time-series or lag-based models were not applied, which may limit sensitivity for detecting delayed short-term responses. However, tournament exposure was temporally dense and overlapping, particularly during periods with multiple matches per day and consecutive match days. Moreover, individual exposure timing was unavailable, and laboratory sampling was clinically driven rather than scheduled relative to matches. Therefore, formal lag-based models would have been difficult to interpret reliably, and short-term effects were assessed using predefined temporal contrasts, including match-day, time-of-day, prime-time, and tournament-phase analyses.

Finally, the study was conducted within a single large tertiary care center. While this provides high data consistency, generalizability to other healthcare systems (e.g., primary care, decentralized networks) or populations with different healthcare utilization patterns may be limited. Additionally, findings may not extrapolate to cultural contexts with markedly different alcohol consumption norms or football engagement levels, as Austria exhibits moderate drinking patterns and high tournament interest.

## 5 Conclusion

In this large real-world analysis, major international football tournaments were not associated with clinically meaningful changes in routine laboratory parameters related to liver function, inflammation, or pancreatic activity. While behavioral changes during such events are well documented, their impact does not appear to translate into measurable biochemical alterations at the population level.

These findings highlight the complexity of linking mass social events to physiological outcomes and support the use of large-scale laboratory data as a complementary tool for public health surveillance. Beyond documenting null effects, this study contributes methodologically by delineating routine lab data’s detection limits for mass gathering impacts and epidemiologically by evidencing population buffering, where behavioral shifts fail to produce aggregate biochemical signals.

## Data Availability

The individual-level datasets analyzed in this study are not publicly available due to institutional data protection regulations. Aggregated data supporting the conclusions of this article may be made available by the corresponding author upon reasonable request.
