# Supplementary material for: Goals, cheers, and gamma-GT: Do football tournaments affect laboratory parameters?
Source: Front Public Health. 2026 Jun 4;14:1839877. doi: 10.3389/fpubh.2026.1839877 (PMC13275681; doi:10.3389/fpubh.2026.1839877)
Supplement: Supplementary file 1 [file Table_1.docx]

| **Supplementary Table 1. Tournament periods and reference windows** |
| --- |

| **Tournament** | **Year** | **Host country / region** | **Reference period (pre)** | **Tournament period** | **Reference period (post)** | **Full observational period** |
| --- | --- | --- | --- | --- | --- | --- |
| **FIFA World Cup** | 2002 | Japan / South Korea | 01.05.2002 – 30.05.2002 | 31.05.2002 – 30.06.2002 | 01.07.2002 – 30.07.2002 | 01.05.2002 – 30.07.2002 |
| **UEFA EURO** | 2004 | Portugal | 13.05.2004 – 11.06.2004 | 12.06.2004 – 04.07.2004 | 05.07.2004 – 03.08.2004 | 13.05.2004 – 03.08.2004 |
| **FIFA World Cup** | 2006 | Germany | 10.05.2006 – 08.06.2006 | 09.06.2006 – 09.07.2006 | 10.07.2006 – 08.08.2006 | 10.05.2006 – 08.08.2006 |
| **UEFA EURO** | 2008 | Austria / Switzerland | 08.05.2008 – 06.06.2008 | 07.06.2008 – 29.06.2008 | 30.06.2008 – 29.07.2008 | 08.05.2008 – 29.07.2008 |
| **FIFA World Cup** | 2010 | South Africa | 12.05.2010 – 10.06.2010 | 11.06.2010 – 11.07.2010 | 12.07.2010 – 10.08.2010 | 12.05.2010 – 10.08.2010 |
| **UEFA EURO** | 2012 | Poland / Ukraine | 09.05.2012 – 07.06.2012 | 08.06.2012 – 01.07.2012 | 02.07.2012 – 31.07.2012 | 09.05.2012 – 31.07.2012 |
| **FIFA World Cup** | 2014 | Brazil | 13.05.2014 – 11.06.2014 | 12.06.2014 – 13.07.2014 | 14.07.2014 – 12.08.2014 | 13.05.2014 – 12.08.2014 |
| **UEFA EURO** | 2016 | France | 11.05.2016 – 09.06.2016 | 10.06.2016 – 10.07.2016 | 11.07.2016 – 09.08.2016 | 11.05.2016 – 09.08.2016 |
| **FIFA World Cup** | 2018 | Russia | 15.05.2018 – 13.06.2018 | 14.06.2018 – 15.07.2018 | 16.07.2018 – 14.08.2018 | 15.05.2018 – 14.08.2018 |
| **UEFA EURO** | 2021 | Pan-European | 12.05.2021 – 10.06.2021 | 11.06.2021 – 11.07.2021 | 12.07.2021 – 10.08.2021 | 12.05.2021 – 10.08.2021 |
| **FIFA World Cup** | 2022 | Qatar | 21.10.2022 – 19.11.2022 | 20.11.2022 – 18.12.2022 | 19.12.2022 – 17.01.2023 | 21.10.2022 – 17.01.2023 |
| **UEFA EURO** | 2024 | Germany | 15.05.2024 – 13.06.2024 | 14.06.2024 – 14.07.2024 | 15.07.2024 – 13.08.2024 | 15.05.2024 – 13.08.2024 |

Overview of all included international football tournaments and predefined observation windows. For each tournament, the event period was defined as the full tournament duration. Reference periods comprised the 30 calendar days before tournament start and the 30 calendar days after tournament end. The full observational period represents the combined pre-tournament reference, tournament, and post-tournament reference windows.
