# Supplementary material for: Goals, cheers, and gamma-GT: Do football tournaments affect laboratory parameters?
Source: Front Public Health. 2026 Jun 4;14:1839877. doi: 10.3389/fpubh.2026.1839877 (PMC13275681; doi:10.3389/fpubh.2026.1839877)
Supplement: Supplementary file 2 [file Table_2.docx]

**Supplementary Table 2. Laboratory parameters on match days versus non-match days within event periods, stratified by clinical setting**

| **Parameter** | **Setting** | **n (No Matchday)** | **Median (IQR) No Matchday** | **n (Matchday)** | **Median (IQR) Matchday** | **Δ (absolute; Matchday- No Matchday)** |
| --- | --- | --- | --- | --- | --- | --- |
| **ALAT** | ED | 3586 | 0.54 (0.40–0.80) | 12597 | 0.54 (0.40–0.80) | 0 |
| **ALAT** | OTHER | 23549 | 0.54 (0.38–0.80) | 71185 | 0.54 (0.40–0.80) | 0 |
| **ASAT** | ED | 3536 | 0.60 (0.48–0.77) | 12448 | 0.60 (0.48–0.77) | 0 |
| **ASAT** | OTHER | 23239 | 0.57 (0.46–0.74) | 70053 | 0.57 (0.46–0.74) | 0 |
| **GGT** | ED | 3126 | 0.53 (0.35–0.97) | 11145 | 0.52 (0.35–0.95) | -0.02 |
| **GGT** | OTHER | 23072 | 0.57 (0.37–1.07) | 69695 | 0.57 (0.37–1.08) | 0 |

Laboratory parameters are presented as ratios to the upper limit of normal (ULN), summarized as median values with interquartile range (IQR) and number of observations (n). Analyses were restricted to event periods and stratified by clinical setting (emergency department [ED] and other ambulatory care). Match days were defined as calendar days within tournament periods on which at least one match was played. Absolute differences (Δ) represent median differences between match days and non-match days.
