# Supplementary material for: Goals, cheers, and gamma-GT: Do football tournaments affect laboratory parameters?
Source: Front Public Health. 2026 Jun 4;14:1839877. doi: 10.3389/fpubh.2026.1839877 (PMC13275681; doi:10.3389/fpubh.2026.1839877)
Supplement: Supplementary file 3 [file Table_3.docx]

**Supplementary Table 3. Laboratory parameters stratified by time of day in event and reference periods**

| **Parameter** | **Time Point** | **n (Reference)** | **Reference (median and IQR)** | **n (Event)** | **Event (median and IQR)** | **Δ (absolute; Event- Reference)** |
| --- | --- | --- | --- | --- | --- | --- |
| **ALAT** | Night | 74669 | 22.00 (16.00–31.00) | 24930 | 21.00 (15.00–31.00) | -1 |
| **ALAT** | Morning | 179030 | 22.00 (16.00–32.00) | 74313 | 22.00 (16.00–32.00) | 0 |
| **ALAT** | Afternoon | 68550 | 21.00 (16.00–32.00) | 42534 | 22.00 (16.00–32.00) | 1 |
| **ALAT** | Evening | 44680 | 21.00 (16.00–32.00) | 55610 | 21.00 (15.00–31.00) | 0 |
| **ASAT** | Night | 74669 | 23.00 (19.00–32.00) | 24930 | 24.00 (20.00–32.00) | 1 |
| **ASAT** | Morning | 179030 | 24.00 (19.00–31.00) | 74313 | 24.00 (19.00–31.00) | 0 |
| **ASAT** | Afternoon | 68550 | 24.00 (19.00–31.00) | 42534 | 24.00 (20.00–32.00) | 0 |
| **ASAT** | Evening | 44680 | 25.00 (20.00–32.00) | 55610 | 24.00 (20.00–32.00) | -1 |
| **CRP** | Night | 74669 | 0.38 ( 0.13– 1.54) | 24930 | 0.42 ( 0.14– 1.40) | 0.04 |
| **CRP** | Morning | 179030 | 0.31 ( 0.12– 0.87) | 74313 | 0.31 ( 0.12– 0.88) | 0 |
| **CRP** | Afternoon | 68550 | 0.34 ( 0.12– 1.13) | 42534 | 0.37 ( 0.13– 1.23) | 0.03 |
| **CRP** | Evening | 44680 | 0.50 ( 0.15– 2.14) | 55610 | 0.55 ( 0.16– 2.37) | 0.05 |
| **GGT** | Night | 74669 | 21.00 (14.00–41.00) | 24930 | 21.00 (13.00–38.00) | 0 |
| **GGT** | Morning | 179030 | 28.00 (17.00–54.00) | 74313 | 28.00 (17.00–54.00) | 0 |
| **GGT** | Afternoon | 68550 | 25.00 (16.00–48.00) | 42534 | 26.00 (16.00–49.00) | 1 |
| **GGT** | Evening | 44680 | 22.00 (14.00–43.00) | 55610 | 22.00 (14.00–40.00) | 0 |
| **KREA** | Night | 711 | 0.87 ( 0.71– 1.03) | 2425 | 0.85 ( 0.70– 1.04) | -0.02 |
| **KREA** | Morning | 19875 | 0.95 ( 0.78– 1.24) | 60795 | 0.95 ( 0.78– 1.22) | 0 |
| **KREA** | Afternoon | 7229 | 0.89 ( 0.73– 1.07) | 22727 | 0.88 ( 0.74– 1.07) | -0.01 |
| **KREA** | Evening | 1648 | 0.89 ( 0.72– 1.09) | 5580 | 0.88 ( 0.73– 1.08) | -0.01 |
| **WBC** | Night | 74669 | 9.37 ( 7.33–11.93) | 24930 | 9.33 ( 7.29–11.97) | -0.04 |
| **WBC** | Morning | 179030 | 6.41 ( 4.92– 8.35) | 74313 | 6.47 ( 4.98– 8.41) | 0.06 |
| **WBC** | Afternoon | 68550 | 7.11 ( 5.59– 9.10) | 42534 | 7.20 ( 5.59– 9.26) | 0.09 |
| **WBC** | Evening | 44680 | 9.01 ( 7.04–11.54) | 55610 | 8.90 ( 6.92–11.39) | -0.11 |

Laboratory parameters are presented as median values with interquartile range (IQR) and number of observations (n) for event and reference periods. Absolute differences (Δ) represent median differences between event and reference measurements.
Time of day was categorized based on sample collection time as follows: night (00:00–05:59), morning (06:00–11:59), afternoon (12:00–17:59), and evening (18:00–23:59).
