# Supplementary material for: Goals, cheers, and gamma-GT: Do football tournaments affect laboratory parameters?
Source: Front Public Health. 2026 Jun 4;14:1839877. doi: 10.3389/fpubh.2026.1839877 (PMC13275681; doi:10.3389/fpubh.2026.1839877)
Supplement: Supplementary file 4 [file Table_4.docx]

**Supplementary Table 4. Laboratory parameters during group stage versus knockout stage, stratified by time of day (prime time vs non–prime time)**

| **Parameter** | **Time Point** | **Group Stage** | **Group Stage (median and IQR)** | **Knockout Stage** | **Knockout Stage (median and IQR)** | **Δ median (Knockout − Group)** |
| --- | --- | --- | --- | --- | --- | --- |
| **ALAT** | Non Prime Time | 52996 | 22.00 (16.00–32.00) | 26940 | 22.00 (16.00–32.00) | 0 |
| **ALAT** | Prime Time | 2447 | 21.00 (15.00–30.00) | 1518 | 21.00 (15.00–32.00) | 0 |
| **ASAT** | Non Prime Time | 52259 | 24.00 (19.00–31.00) | 26545 | 24.00 (19.00–31.00) | 0 |
| **ASAT** | Prime Time | 2399 | 24.00 (20.00–31.00) | 1494 | 25.00 (20.00–34.00) | 1 |
| **GGT** | Non Prime Time | 51294 | 27.00 (17.00–52.00) | 26010 | 27.00 (17.00–51.00) | 0 |
| **GGT** | Prime Time | 2222 | 22.00 (14.00–39.00) | 1404 | 22.00 (14.00–44.00) | 0 |

Laboratory parameters are presented as median values with interquartile range (IQR) and number of observations (n) for group stage and knockout stage periods. Absolute differences (Δ) represent median differences between knockout stage and group stage measurements.
Prime time was defined as blood sample collection between 18:00 and 22:00, while non–prime time comprised all remaining time periods.
