# Supplementary material for: Goals, cheers, and gamma-GT: Do football tournaments affect laboratory parameters?
Source: Front Public Health. 2026 Jun 4;14:1839877. doi: 10.3389/fpubh.2026.1839877 (PMC13275681; doi:10.3389/fpubh.2026.1839877)
Supplement: Supplementary file 5 [file Table_5.docx]

**Supplementary Table 5. Laboratory parameters during Austria matches versus other matches, stratified by time of day (prime time vs non–prime time)**

| **Parameter** | **Time Point** | **Other Nation Matches** | **Other Nation Matches (median and IQR)** | **Austria Matches** | **Austria Match (median and IQR)** | **Δ (absolute; Austria Match- Other Nation Matches)** |
| --- | --- | --- | --- | --- | --- | --- |
| **ALAT** | Non Prime Time | 76274 | 22.00 (16.00–32.00) | 3662 | 22.00 (16.00–32.00) | 0 |
| **ALAT** | Prime Time | 3780 | 21.00 (15.00–31.00) | 185 | 22.00 (15.00–32.00) | 1 |
| **ASAT** | Non Prime Time | 75160 | 24.00 (19.00–31.00) | 3644 | 24.00 (19.00–31.00) | 0 |
| **ASAT** | Prime Time | 3707 | 25.00 (20.00–32.00) | 186 | 24.00 (20.00–33.00) | -1 |
| **GGT** | Non Prime Time | 73778 | 27.00 (17.00–52.00) | 3526 | 26.00 (16.00–50.00) | -1 |
| **GGT** | Prime Time | 3445 | 22.00 (14.00–40.00) | 181 | 21.00 (14.00–40.00) | -1 |

Laboratory parameters are presented as median values with interquartile range (IQR) and number of observations (n) for Austria matches and other matches within tournament periods. Absolute differences (Δ) represent median differences between Austria matches and other matches.
Prime time was defined as blood sample collection between 18:00 and 22:00, while non–prime time comprised all remaining time periods.
